# Improvement in nanofat preparation technology: Simple and easy-to-use adipose tissue harvesting with Liporevive

**DOI:** 10.1016/j.jpra.2025.09.019

**Published:** 2025-09-21

**Authors:** Ekaterina V. Semina, Zoya I. Tsokolaeva, Dmitry S. Katserov, Alexander S. Prokoptsov, Alexander B. Tristanov, Sergey V. Kruglik, Ekaterina V. Davtyan, Viktor V. Kakotkin

**Affiliations:** a Institute of Medicine and Life Science, Immanuel Kant Baltic Federal University, Kaliningrad, Russia; b Negovsky Scientific Research Institute of General Reanimatology of the Federal Research and Clinical Center of Intensive Care Medicine and Rehabilitology, Moscow, Russia; c Digital Technology Institute, Kaliningrad State Technical University, Kaliningrad, Russia; d Department of Plastic Surgery, VIP Clinic, Kaliningrad, Russia; e Department of Plastic Surgery and Cosmetology, SVS Clinic, Moscow, Russia

**Keywords:** Liporevive, Nanofat, Adipose tissue, Mechanical disaggregation, Reconstructive surgery, Regenerative medicine

## Abstract

**Background:**

Autologous adipose tissue is widely used in clinical practice as a favorable material for soft tissue filling. Despite the range of approaches for its processing the main aim is investigating a universal and simple method for processing lipoaspirate to obtain nanofat with optimal histological characteristics and cellular composition.

**Methods:**

Lipoaspirates obtained from the abdominal fat were analyzed. Microfat was obtained by Luer-to-Luer Syringe adapter. Mechanical disaggregation of lipoaspirates were obtained by Liporevive equipped with stainless steel meshes. Using 30 or 50 filtrations through 1.4-, 0.8-, 0.6- and 0.4-mm pore size meshes, nanofat-containing samples were further analyzed for the presence of lipids, endothelial and stromal cells and DNA concentration.

**Results:**

The Liporevive-obtained nanofat samples resembled the tissue structure of the microfat sample and revealed non-damaged parts of tissue filled with intact adipocytes. After using meshes with parameters (input/output/number of times) of 1.4/0.6/30, 1.4/0.4/30, 0.8/0.6/30, 1.4/0.6/50 and 1.4/0.4/50, nanofat samples with DNA concentrations similar to microfat were obtained. Decreasing the mesh’s pore sizes to 0.4 mm (output) and increasing the number of filtrations to 50 reduced the content of fat droplets in the nanofat samples, as well as stromal, but not in the endothelial cells.

**Conclusion:**

Mechanical disaggregation of lipoaspirate 30 times with Liporevive, using meshes with a pore diameter of 1.4 mm at the input and 0.6 mm at the output, allows obtaining nanofat comparable to lipoaspirate in DNA concentration, stromal, endothelial and adipose-derived stem cells — key components of regenerative potential. The advantage of Liporevive is obtaining nanofat without additional enzyme treatments.

## Introduction

Mechanical and enzymatic processing of lipoaspirate to obtain nanofat or stromal-vascular fraction (SVF) are the main methods for obtaining adipose tissue-based autograft with regenerative properties. Enzymatic processing of lipoaspirate allows obtaining a cell’s fractions with high viability and properties. However, a higher operator involvement, speed and cost necessitate investigating a simpler method of emulsification and disaggregation of adipose tissue. Using meshes (Tulip Medical Products, LipoCube™ Nano, Adinizer) or stainless steel marbles (Lipogems)^1^ for lipoaspirate disaggregation allows the increase of tissue homogeneity from low for microfat to high for nanofat, considered as SVF^2^ since it contains a large number of colony-forming stromal cells with multi-differentiation potential.

The biological properties of the nanofat depend on the fat tissue structure preservation, stromal and vascular cells appearance and the extracellular matrix content. Decreasing the pores’ diameter in emulsifying meshes resulted in reducing volume-forming properties of micro- or nanofat samples, although the regenerative potential remains comparable due to the preservation of stromal cells that are more resistant to mechanical damage.^3^ Using techniques that combine the ease of obtaining nanofat with maximum preservation of biologically active material (cells and matrix) requires evaluating methods of mechanical processing of adipose tissue. Existing systems allow obtaining separated fractions of microfat and nanofat in one manipulation with adipose tissue, as it allows filling large and small defects in one approach, for use in rejuvenation purposes and for treating deep wounds. The question of selecting the filtration rate through separation networks is open due to insufficient research and a conservative approach to its assessment.

Characteristics of the final product after mechanical processing of lipoaspirate is assessed by describing the morphology (histology, cytology),^4^, ^5^, ^6^ assessing the qualitative and quantitative cellular composition (flow cytometry)^7^ and determining the viability of nucleated cells.^5^^,^^8^^,^^9^

Here, using the Liporevive - closed sterile system for mechanical disaggregation of lipoaspirate to obtain nanofat, we compared characteristics of nanofat with lipoaspirate or microfat. We have shown that Liporevive is a simple technique that allows obtaining nanofat samples without significant DNA loss compared to lipoaspirate and microfat, and contains stromal and vascular cells (the tissue retains its cellular microarchitecture) and Adipose-Derived Stem Cells (ADSCs) which have regenerative capacity. The best method for obtaining nanofat using Liporevive is using separation meshes with 1.4 mm pore sizes at the input and 0.6 mm at the output and 30 filtrations successively through each of them.

## Materials and methods

### Ethic

The protocol was conducted in accordance with the World Medical Association Declaration of Helsinki “Ethical Principles for Conducting Medical Research Involving Human Subjects” (rev. in 2013) and the Rules of Good Clinical Practice in the Russian Federation (June 19, 2003, Registration No 266).

The protocol of the study was approved by Ethics Board of VIP Clinic (Approval letter, October 12, 2023). Informed consents were received from all participants prior to inclusion in the study.

### Patients

The study included 10 fat samples of 10 donors aspirated under low negative pressure from the lower abdomen under endotracheal anesthesia. The most commonly used source for micro/nanofat obtaining is lipoaspirate harvested from the abdomen and the lateral thigh. This specific localization was chosen due to the characteristics of the population. Among the patients meeting the inclusion criteria, the overwhelming majority sought abdominal liposuction. To ensure uniformity of samples during the statistical analysis of the results, only adipose tissue samples obtained from the lower abdomen were utilized. Exclusion criteria: diabetes mellitus or other endocrine diseases, immunosuppressive or anticancer therapy, presence of HIV, chronic hepatitis C, active bacterial infection. All participants were cisgender Caucasian women; the median age was 37 years (the min - 26 years, the max - 48 years).

The median BMI before surgery was 28.4 kg/m^2 (minimum - 25.3 kg/m^2, maximum - 34.2 kg/m^2), and after surgery - 27.5 kg/m^2 (minimum - 25.0 kg/m^2, maximum - 31.7 kg/m^2).

### Adipose tissue harvesting and mechanical disaggregation of lipoaspirate

A solution containing 500 ml of physiological saline, 2 ml of 10 % lidocaine and 1 ml of 0.01 % adrenaline was injected into the extraction area for 20 min. A 60 ml syringe with a vacuum lock was used to generate a vacuum; the diameter of cannula - 2.41 mm. All samples were delivered to the laboratory within 2–3 h for further analysis.

**Figure 1:**
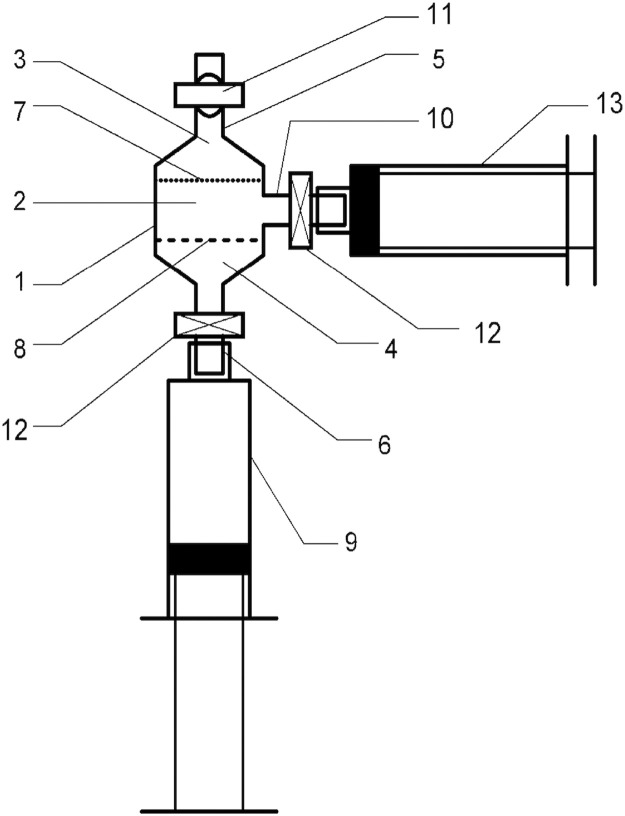
The structural characteristics of the Liporevive. The device comprises: a body (1), the middle part (2) of which has a cylindrical shape, and the upper (3) and lower (4) are tapered, passing into channels with fittings (5) and (6). Inside the body (1), mesh elements (7) and (8) with different hole sizes are fixed: the upper (7) with a cell size of 0.4 mm, the lower (8) with a size of 1.5 mm. Between the mesh elements (7) and (8) free space (2) is located. Valve (11) is installed on the nipple (5). In the middle part of the body (1), an additional nozzle (10) is installed perpendicular to the axis of the device. Nozzles (6) and (10) are equipped with a hermetically closed valve (12) in a free state and automatically switches to an open state when a syringe is attached to it.

**Figure 2:**
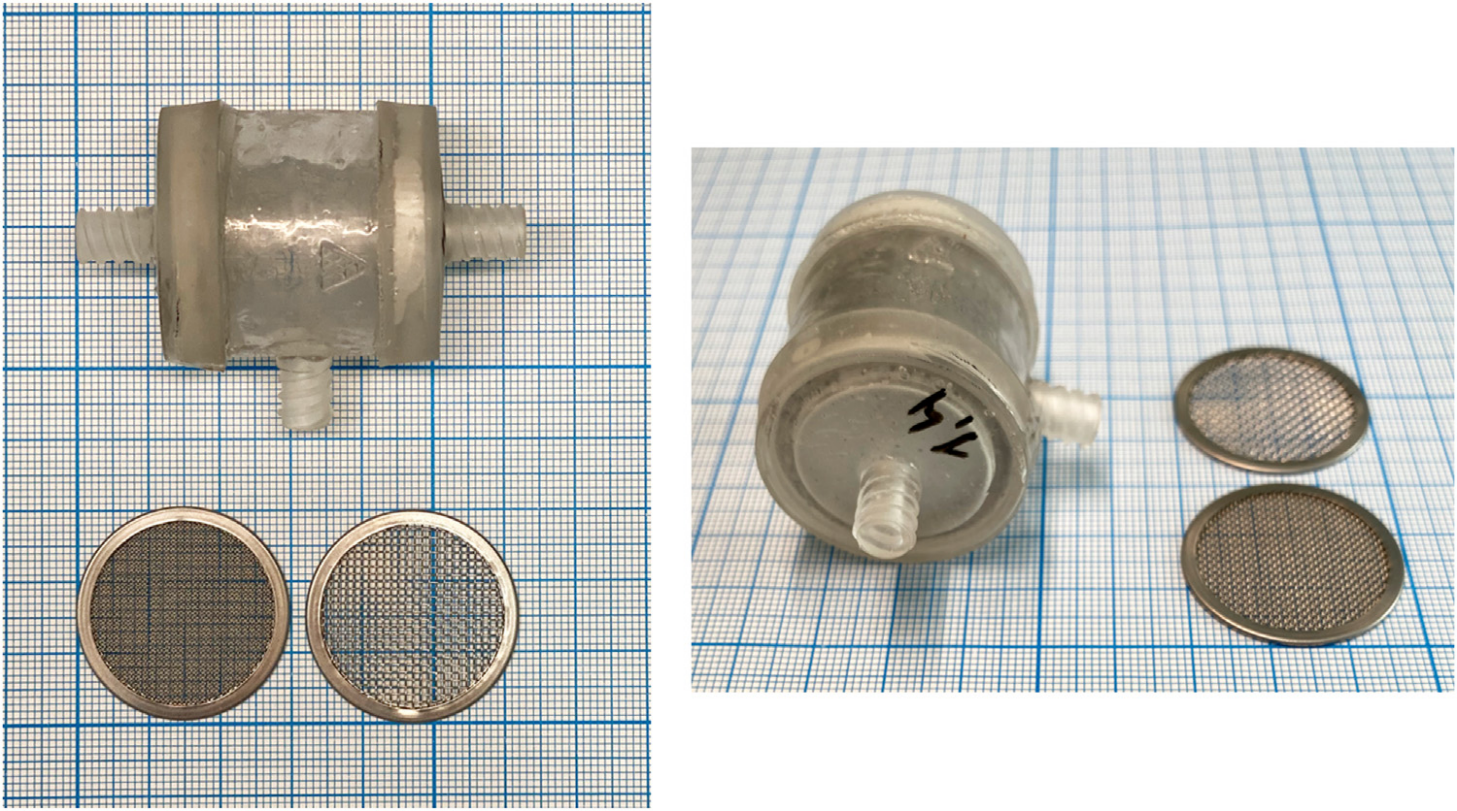
Form and characteristics of the Liporevive and meshes. One small division on the scale corresponds to 0.5 mm.

Mechanical emulsification of lipoaspirate was passed through Liporevive - closed sterile anaerobic system for adipose tissue processing (Figure 1).^10^ Liporevive entails emulsifying stainless steel mesh inserts (AISI 420) with 1.4-, 0.8-, 0.6- and 0.4-mm pore size, which allows to obtain nanofat.^8^ 3D Printing and Photopolymer Anycubic Basic Clear resin were used for device formation (Figure 2).

**Figure 3:**
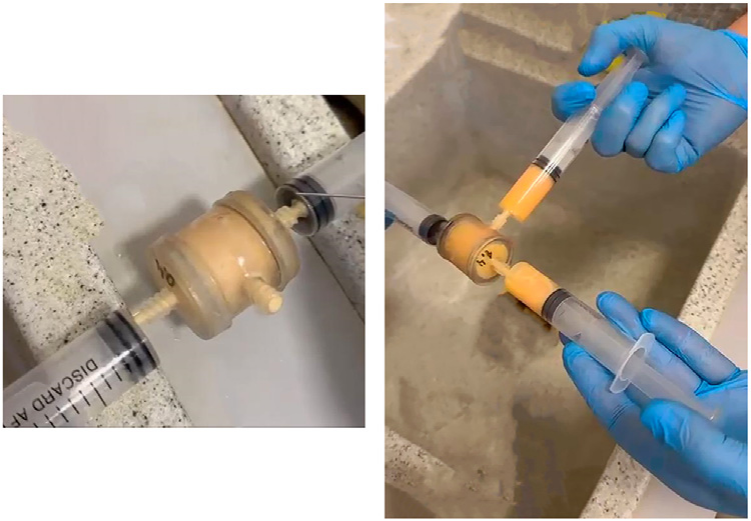
Obtaining nanofat Samples (S3 - S7) by the Liporevive - a single-use approach for mechanical processing of lipoaspirate or autologous fat tissue into nanofat grafts.

Obtaining nanofat samples included filtrations using 20-ml syringes via one mesh with bigger pore sizes, further equivalent filtrations via mesh with smaller pore sizes (Figure 3).

Sample description (see detailed description in Supplementary materials 2):

• S1: untreated lipoaspirate.
• S2: microfat, passed 30 times through a 1.4 mm Luer-to-Luer adapter.
• S3-S7: nanofat fractions processed via Liporevive by sequentially passing lipoaspirate through meshes of varying pore sizes and repetitions:
  ○ S3: 30 × through 1.4 mm mesh, then 30 × through 0.6 mm mesh.
  ○ S4: 30 × through 1.4 mm, then 30 × through 0.4 mm mesh.
  ○ S5: 30 × through 0.8 mm, then 30 × through 0.6 mm mesh.
  ○ S6: 50 × through 1.4 mm, then 50 × through 0.6 mm mesh.
  ○ S7: 50 × through 1.4 mm, then 50 × through 0.4 mm mesh.

All emulsified adipose tissue appeared whitish; nanofat samples (S3-S7) formed homogeneous emulsions. Samples were split into two parts: one for unfixed smears (S2-S7) prepared on SuperFrost Plus© slides, fixed in 10 % formalin, and stained; the other fixed in 10 % formalin (1:2 ratio), paraffin-embedded, sectioned at 2 µm (S1-S7), and stained as described.

### Immunohistochemistry and microscopy

Formalin-fixed, paraffin-embedded 2 µm sections (Samples S1-S7) were deparaffinized and rehydrated. Unfixed smears (S2-S7) were fixed in 10 % formalin and rinsed in PBS. Antigen retrieval was performed using Trilogy buffer (Sigma-Aldrich) per manufacturer’s instructions. Endogenous peroxidase was blocked with 3 % hydrogen peroxide for 20 min, followed by blocking with 1 % BSA/PBS for 30 min at room temperature. Primary antibodies against vWF (Abcam ab6994), ɑ-SMA (Abcam ab5694), or CD73 (Abcam ab175396) were applied at 1:100 dilution for 1 h. Real EnVision HRP-conjugated secondary antibodies (Dako) were applied for 30 min. Slides were washed thrice with PBS, incubated with HiDef Detection™ HRP Polymer Detector (Cell Marque) for 10 min, and developed with DAB substrate (Dako) for 2 min. After stopping the reaction with distilled water, slides were dehydrated, cleared, and mounted with Cytoseal™ 60 (Thermo Scientific). H&E staining was performed by standard protocol. Slides were scanned using Aperio ImageScope (v12.4.3.5008, Leica Microsystems). See Supplementary Materials 2 for detailed description.

### Lipids staining and microscopy

Unfixed smears (S2-S7) were fixed in 10 % formalin, rinsed with PBS, dehydrated in 70 % ethanol, and stained with Sudan III (Biovitrum) for 30 min. Slides were washed in 70 % ethanol and PBS, counterstained with H&E, and mounted with Aqua-Poly/Mount (PolySciences). Images were captured using Aperio ImageScope (v12.4.3.5008). Sudan III staining area was quantified using the Positive Pixel Count v9 algorithm, calculating the staining score as the ratio of positive pixels to total pixels (Np / (Np + Nn)). See Supplementary Materials 2 for detailed description.

### Evaluation of DNA quantity in samples

Samples were lysed with buffer and beads, incubated and homogenized at 90 °C, then centrifuged and extracted with chloroform/isoamyl alcohol. DNA was precipitated with NaCl and ethanol at −20 °C overnight, centrifuged, washed with ethanol, and dried before quantification using Qubit. See Supplementary Materials 2 for detailed description.

### Statistical analysis

One-way analysis of variance (ANOVA) was used to compare the groups and determine significance between groups. The Tukey's test was used further to identify groups significantly different from each other. Data was analyzed using the python scipy.stats module. Data is presented as median, IQR, 25th-75th percentile; the level of significance was set at 0.05*.*

## Results

**Figure 4:**
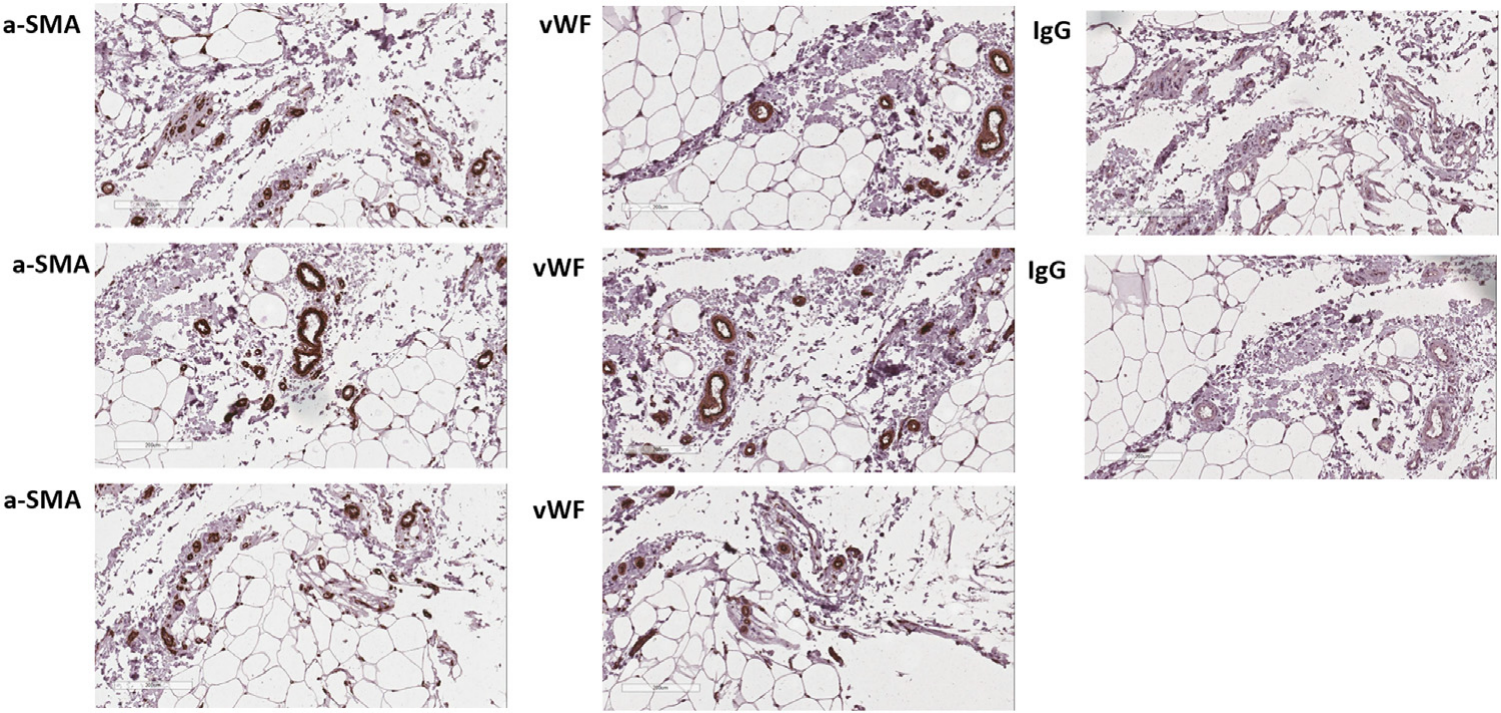
Immunohistochemical assessment of stromal and endothelial cells in biopsy of adipose tissue. Three of each representative sections stained for stromal cell marker a-SMA (left panel) and endothelial-cell marker vWF (central panel) were revealed with DAB chromogen substrate (brown); non-immune IgG was used as a control (right panel). The slides were counterstained with hematoxylin (blue). Presented representative photomicrographs of donor-independent staining. The scale bar is 200 µm.

The goal of this study was to compare the Samples obtained by Liporevive with naïve fat, lipoaspirate, and minimally processed lipoaspirate (microfat). Initially, ICH staining of naïve fat revealed adipocytes, endothelial and stromal cells (Figure 4).

**Figure 5:**
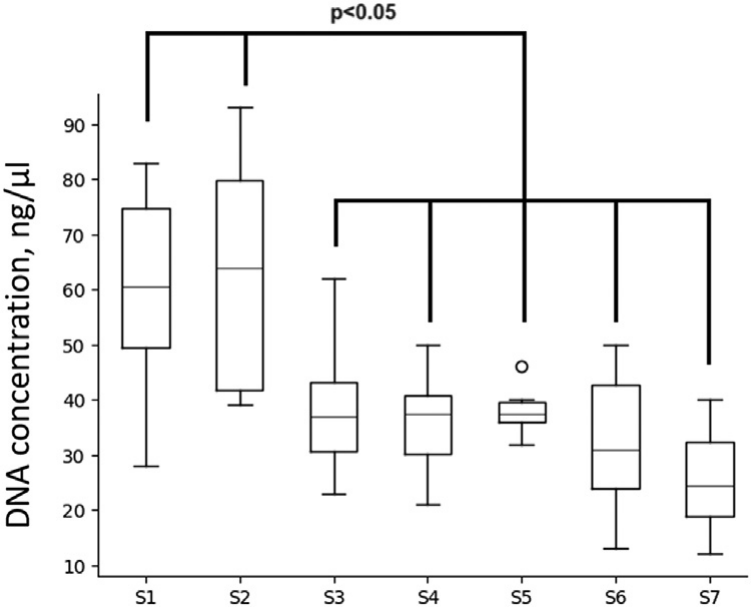
DNA concentration in Samples. Sample S1 corresponds to lipoaspirate, S2- microfat. Samples S3-S7 correspond to nanofat. Data is presented as median, IQR, 25th-75th percentile; the level of significance was set at *p* < 0.05.

We demonstrated decline in DNA concentration in all nanofat Samples (S3-S7) compared to lipoaspirate S1 and microfat S2 (Figure 5), however, no significant difference was found between nanofat S3-S7; generally, all Samples contained comparable DNA concentrations. Thus, selecting the parameters for nanofat (S3-S7) obtained by Liporevive (meshes and filtration) doesn’t affect further cell destruction more than when it occurs during obtaining of nanofat from lipoaspirate (S1) or microfat (S2).

**Figure 6:**
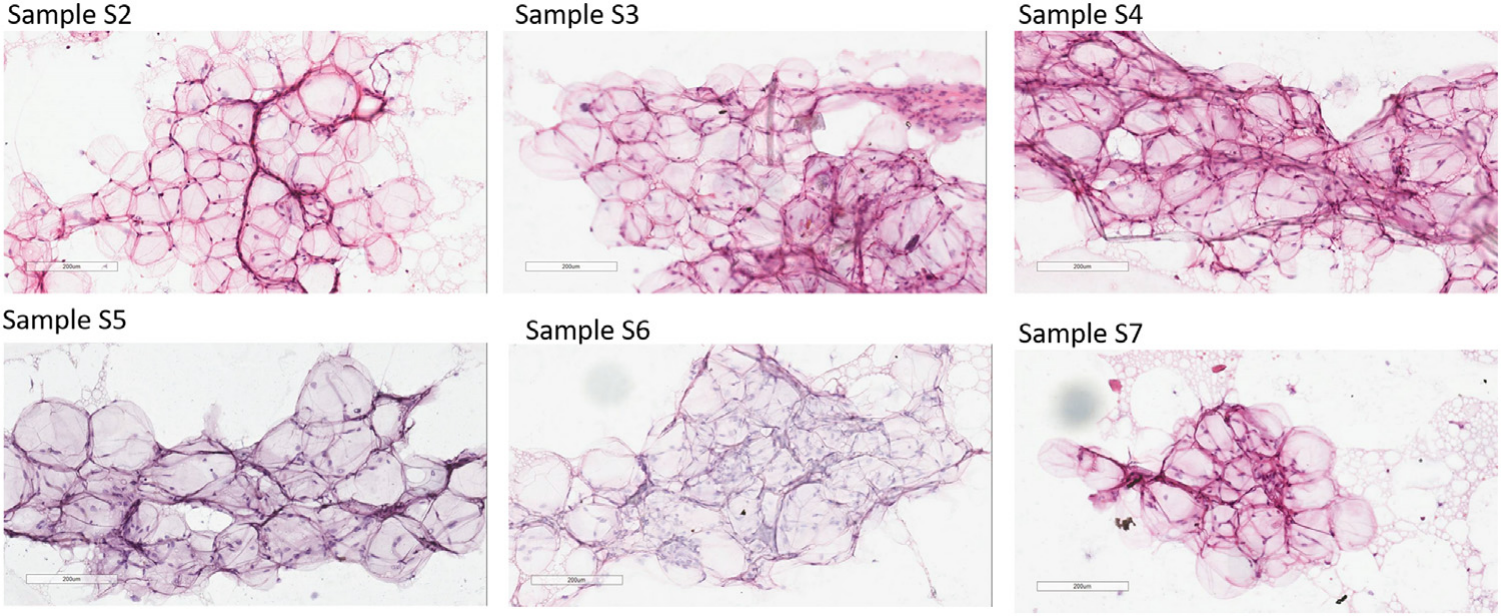
Hematoxylin-Eosin staining of Samples’ smears. S2 corresponds to microfat and S3-S7 corresponds to nanofat. Sample S1 is not provided due to inability to make a lipoaspirate smear. All Samples contain aggregates of adipocytes with remnants of connective tissue; a large number of morphologically intact adipocytes appears. Sample S7 contains the elements of the fat. Representative data of donor-independent staining are presented. The scale bar is 200 µm.

Histologically the Liporevive-obtained nanofat S3-S7 showed non-damaged parts of tissue filled with intact adipocytes (show the general structure of fat cells) and resembled the tissue structure of the microfat sample S2 (Figure 6). H&E staining Samples’ smears didn’t reveal significant changes in the tissue’s structure between microfat and nanofat (Samples S2-S7) and within different processes of obtaining nanofat (meshes and filtrations). Sample S1 is not provided due to infeasibility to make a smear of lipoaspirate.

**Figure 7:**
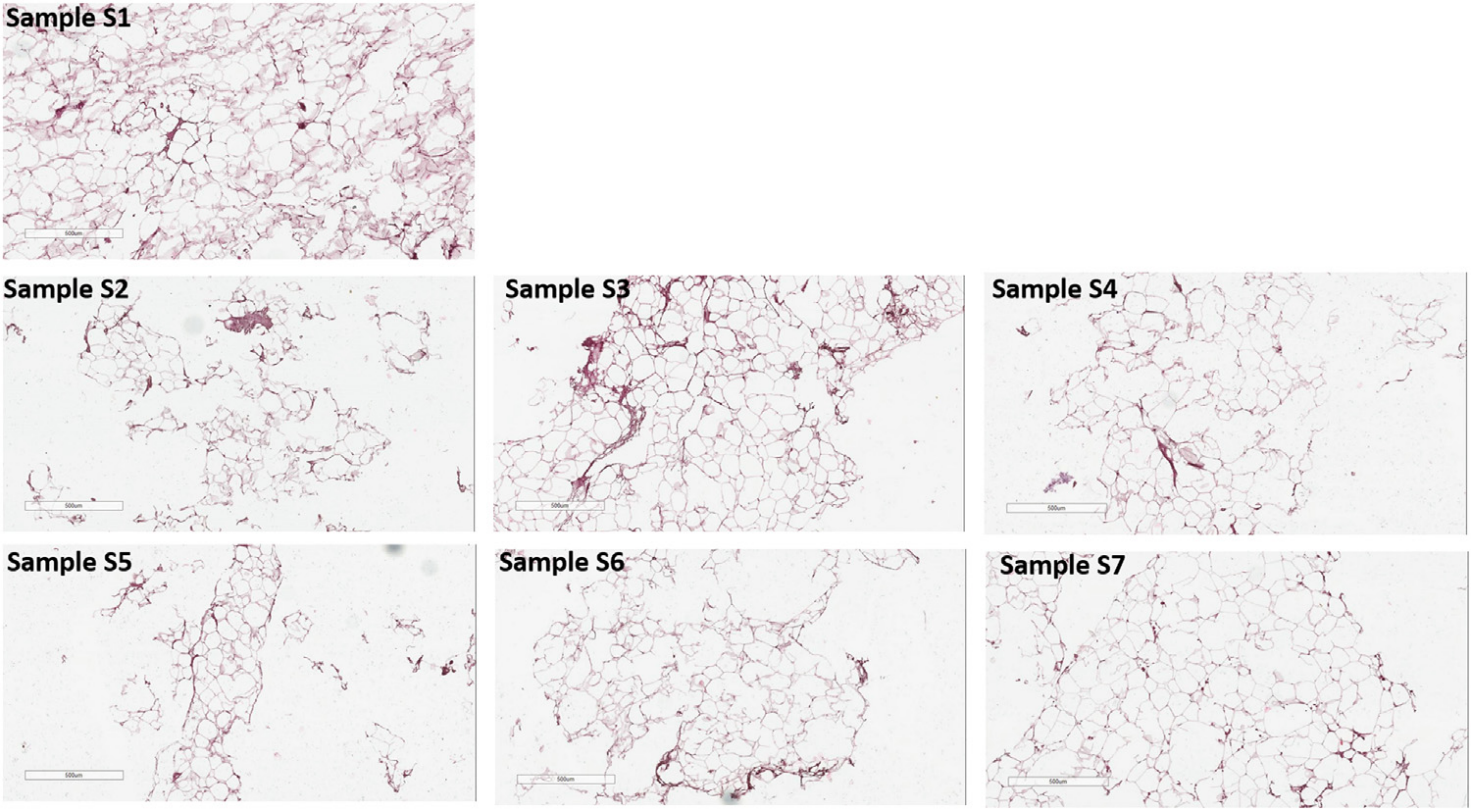
Hematoxylin-Eosin staining of Samples. S1 corresponds to lipoaspirate, S2 corresponds to microfat and S3-S7 corresponds to nanofat. Samples S2-S7 demonstrate destroyed adipocytes and naive adipose tissue (consisting of non-damaged adipocytes and stromal components) simultaneously. Representative data of donor-independent staining is presented. The scale bar is 500 µm.

More precise analysis of 2 µm sections of Samples S1-S7 revealed that nanofat obtaining by Liporevive resulted in partially damaging adipose tissue and adipocytes (S2-S7 Samples, Figure 7) compared to lipoaspirate (S1 Sample, Figure 7), nevertheless intact adipocytes and general adipose tissue still appears.

**Figure 8:**
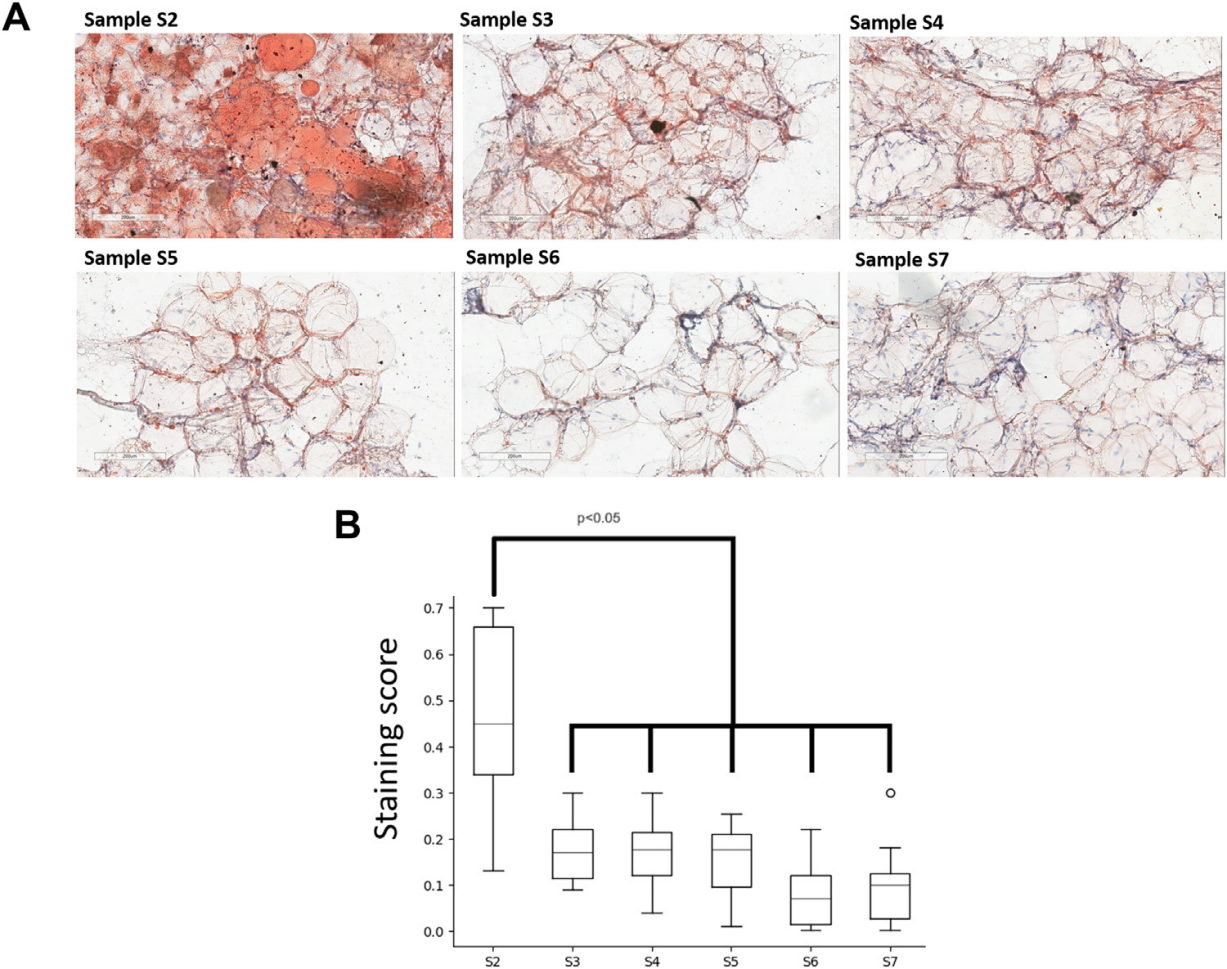
Analysis of lipid content in Samples. (A) Representative images of Sudan III staining of Samples’ smears. Sample S1 is not provided due to impossibility to make a smear of lipoaspirate. S2 corresponds to microfat and S3-S7 corresponds to nanofat. Nanofat Samples S2-S7 are detected with morphologically-related adipocytes and preserved adipose tissue structures. The slides were counterstained with hematoxylin (blue). Representative data of donor-independent staining are presented. The scale bar is 200 µm. (B) Images were analyzed using the Positive Pixel Count v9 algorithm of ImageScope (Aperio), which counts pixels of the predetermined color (orange for Sudan III) and pixels related to other colors (negative pixels). A staining score was calculated as the number of positive pixels/(number of positive + negative pixels). Data is presented as median, IQR, 25th-75th percentile; the level of significance was set at *p* < 0.05.

The Samples’ smears thereafter were stained with Sudan III for fat content analysis (Figure 8A). We detected in the Sample S2, corresponding to microfat, large-sized fat droplets equally distributed throughout the sample. Sample S1 is not provided due to infeasibility to make a smear of lipoaspirate.

In S3-S7 nanofat obtained by Liporevive, the fat content was reduced compared to S2 microfat (diagram in Figure8 B, *p* < 0.05) indicating that processing the microfat resulted in reducing fat content. Simultaneously, the lowest (although non-significant) residual fat content is observed in nanofat S6 and S7, which were obtained after 50 filtrations through 1.4/0.6 and 1.4/0.4 mm meshes, respectively.

**Figure 9:**
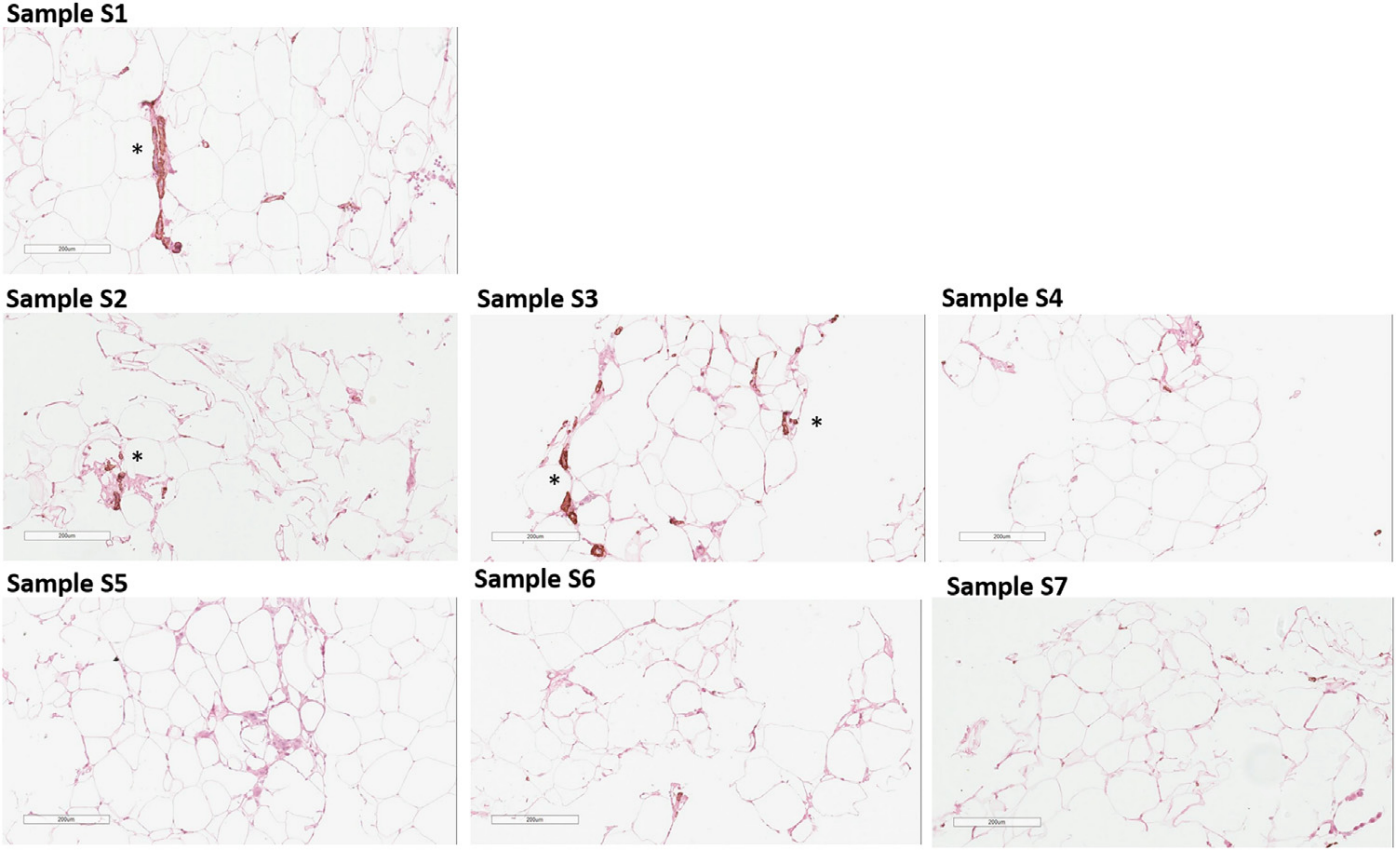
Paraffin-embedded sections were stained to detect stromal cells (a-SMA, brown). Representative images of S1-S7 Samples are shown; asterisks indicate a-SMA-positive staining. The slides were counterstained with hematoxylin (blue). Representative data of donor-independent staining is presented. The scale bar is 200 µm.

Regarding the integrity of cells or structures capable of providing pro-regenerative potential in nanofat, we further assessed the content of stromal and vascular cells in Samples S1-S7. Figure 9 demonstrates ICH staining data of Samples’ sections for a-SMA fibroblasts/stromal cells marker.

S2-S7 Samples showed irregular-sized/shaped adipocytes, which seemed to be damaged or dead. In minimally processed samples (lipoaspirate Sample S1 and microfat S2), structures containing stromal cells are present, however, obtainment of nanofat inevitably leads to decreasing of a-SMA-positive structures, which can be explained by the loss/destruction of stromal cells as a result of mechanical processing. We found a-SMA-positive structures in Sample S3, while their complete absence was observed in nanofat S4-S7.

**Figure 10:**
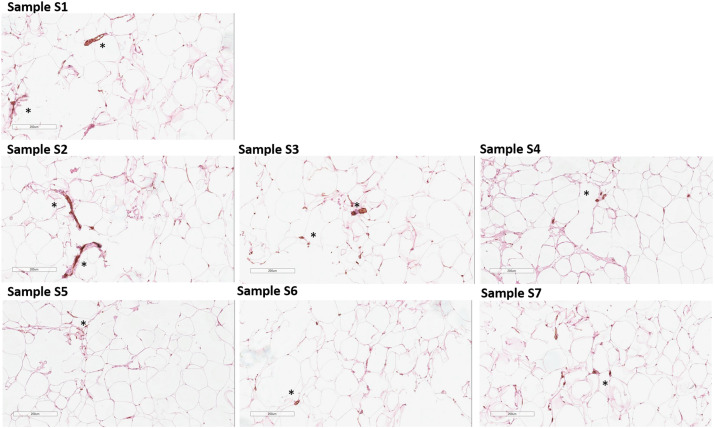
Paraffin-embedded sections were stained to detect endothelial cells (vWF, brown). Representative images of S1-S7 Samples are shown; asterisks indicate vWF-positive staining. The slides were counterstained with hematoxylin (blue). Representative data of donor-independent staining is presented. The scale bar is 200 µm.

Simultaneously, histologic analyses revealed that all of the Samples S1-S7 contain endothelial cells and vessels (Figure 10). Positive staining to a vWF revealed its presence in all Samples.

**Figure 11:**
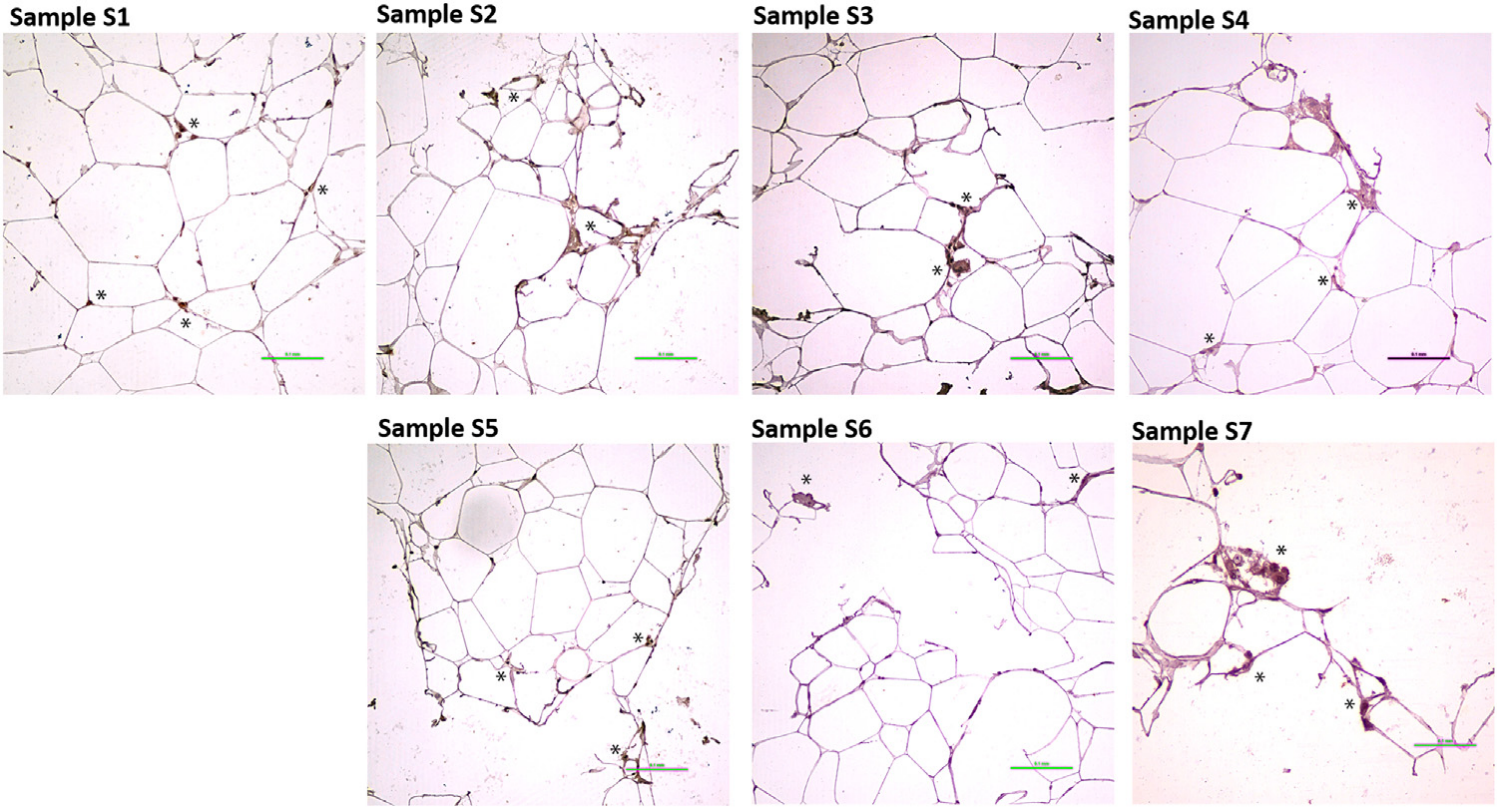
Paraffin-embedded sections were stained to adipose-derived stem cells. Representative images of S1-S7 Samples are shown; asterisks indicate ADSCs-positive brown staining. The slides were counterstained with hematoxylin (blue). Representative data of donor-independent staining is presented. The scale bar is 0.1 mm.

Regarding the expression of the ADSC marker, we further analyzed the presence of CD73 antibodies in all samples.^11^^,^^12^ Figure 11 illustrates the detection of this marker across all samples (S1-S7), indicating that as a result of mechanical processing of adipose tissue through Liporevive, we are able to preserve the fraction of ADSCs that possess pro-regenerative potential. This finding suggests that despite the processing, the essential stromal cell population remains intact, which is crucial for their potential application in regenerative therapies.

Thus, mechanical processing of lipoaspirate via 30 filtrations through meshes (1.4 mm input/0.6 mm output pore diameters) using the Liporevive yields optimally conditioned nanofat demonstrated DNA concentration parity with standard lipoaspirate while preserving stromal, endothelial, and adipose-derived stem cells — key mediators of regenerative potential.

## Discussion

Biomaterials derived from adipose tissue are becoming more widespread in clinical practice. The presence of stem cells in adipose tissue, as well as their preservation in the obtained fat samples based on it, enhance the advancement of adipose tissue-derived products in plastic surgery, rejuvenation applying, treatment of orthopedic diseases, wounds regeneration.^13^^,^^14^ Most common methods for obtaining nanofat are mechanical, using mechanical disaggregation (emulsification) of lipoaspirate, enzymatic isolation, or their combination.^15^ A number of authors have previously hypothesized that nanofat obtained using mechanical devices are not inferior in their effect to preparations of the stromal-vascular fraction.^8^^,^^9^

Considering the small size of the nanofat emulsion, its use is preferably indicated in cases where the main expected effect of autotransplantation is regenerative: for the correction of scars and filling of small defects in soft tissues with an initially low regenerative potential, as well as for aesthetic purposes.^14^ Despite the obvious difficulties in determining the size of the obtained nanofat samples, it is believed that nanofat obtained using meshes, has a size not exceeding 600–800 nm^8^. The meshes’ sizes of the Liporevive (Figure 2) allow the production of fat samples that meet the definition of “nanofat.”

Mechanical disaggregation of adipose tissue allows obtaining nanofat samples with parameters comparable to the lipoaspirate or adipose tissue.^16^^,^^17^ Here, we demonstrated that the DNA concentration in nanofat S3-S7 obtained by Liporevive is comparable to the initial DNA concentration in Sample S1 of lipoaspirate or S2 microfat (Figure 5). In addition, the qualitative composition of nanofat, despite the destruction of adipocytes, still contains stromal cells and vascular cells (Figure 9, Figure 10) that have a pro-regenerative effect.^18^ Published data does not reveal the information about DNA quantification in nanofat including its comparison with lipoaspirate and microfat. Despite the obvious limitation of the DNA comparison in all Samples S1-S7, including lipoaspirate, micro- and nanofat (in particular, the DNA concentration in nanofat samples does not characterize the DNA concentration in intact cells, and also limits the conclusion about the preservation of cells in the samples), its combination with histologic and ICH results is not inferior to cytofluorimetric or viability tests for analyzing the number and integrity of cells.^8^^,^^16^

Mechanical processing offers advantages over enzymatic methods for obtaining nanofat, such as simplicity, speed, and lower contamination risk without needing extra equipment. Although enzymatic or combined methods may be more efficient, their drawbacks, including lengthy fat preparation, longer extraction time, high costs, need for specialized labs or devices, and risk of allergic reactions from residual enzymes, limit their popularity among surgeons.^19^

Using the Liporevive allows the processing of lipoaspirate until nanofat is obtained, with the characteristics of a minimally manipulated product that can be readily injected in an autologous fashion into the donor. The overall procedure is very fast and simple. The highest preservation of adipose tissue is obtained in the S3 Sample (mechanical disaggregation of lipoaspirate 30 times, 1.4 mm input/0.6 mm output). This approach achieves the homogeneous emulsified adipose tissue with a high content of nondamaged parts of adipose tissue filled with morphologically intact adipocytes (Figure 6, Figure 7).

The properties of adipose-derived transplants depend on the emulsification type and preservation of adipocytes and extracellular matrix structure.^13^ As an accessible source of mesenchymal stem cells, fibroblasts, and their precursors, as well as endothelial progenitor cells, subcutaneous adipose tissue is utilized for regenerative purposes. The culture of the ADSCs component generates a population of cells expressing a wide range of markers, including CD73 expression.^20^^,^^21^ As fat fragment diameter decreases, volume-forming ability drops, but regenerative potential stays similar because mesenchymal and stromal cells resist mechanical damage.^3^^,^^22^ The ICH characterization of nanofat obtained through Liporevive revealed the presence of CD73+, α-SMA+, and vWF+ cells within the structure of the Samples (Figure 9, Figure 10, Figure 11), assuming their functioning in tissue repair and regeneration. Expected results has been obtained: a decrease in the size of the meshes and an increase in the number of filtrations reduces the content of endothelial and a-SMA-positive stromal cells in nanofat S3-S7. However, their presence is still detectable (in S3, Figure 9 for a-SMA, in S3-S7 Figure 10, for vWF) and this allows us to conclude about the utilization of nanofat samples obtained by Liporevive for a wide range of clinical applications. As for the distribution of CD73+ cells, Figure 11 demonstrates positive staining across samples S1-S7, demonstrating that mechanical processing of adipose tissue via Liporevive preserves the fraction of ADSCs with pro-regenerative potential.

The method used in our study for formalin-fixed paraffin-embedded Samples’ sections (S1-S7) has an advantage. In particular, in a number of similar studies described, samples are centrifuged or washed before analyzing and a false idea of the “distribution density” of the cellular and stromal component in the obtained samples may be formed.^3^^,^^17^ Our results allow us to estimate the true ratio of stromal and vascular component cells in nanofat obtained by Liporevive.

This study did not compare Liporevive-processed lipoaspirate with other mechanical methods. Instead, we analyzed cellular and stromal components of nanofat obtained using different metal mesh combinations and processing cycles. Bianchi et al. showed^23^ that Lipogems preserves the vascular stromal niche and pericytes similarly to our S3 samples but retains more stromal elements across processing intensities than Liporevive, where α-SMA+ cells decline from S4 to S7. Cohen SR et al. reported^8^ that LipoCube reduces adipocyte integrity while maintaining CD73+ ADSCs, aligning with our findings; however, our 30 filtrations through 1.4/0.6 mm meshes better preserve tissue integrity than LipoCube’s higher passage counts. Tonnard et al. found^24^ Tulip systems decrease adipocyte viability but preserve ADSCs, consistent with our CD73 data, though Tulip requires centrifugation steps absent in Liporevive. These results suggest Liporevive is a promising mechanical processing alternative, pending further direct comparisons.

At present, comparing the costs of the Liporevive method with those of other systems poses a challenge. According to information provided by official distributors, the Lipocube Nano device is priced at approximately 200 USD, while the disposable All in One Lipogems Kit, with a capacity of 240 mL, is priced at around 1700 USD. Additionally, the Tulip Nanofat Set, designed for multiple uses, costs 1510 USD. It is important to note that the Liporevive system does not currently have an established market value, as it has been supplied in limited quantities for testing purposes and has not yet entered serial production.

## Limitations

The study focuses on the *in vitro* analysis of nanofat obtained through the Liporevive, demonstrating its potential applications in tissue regeneration. However, it is necessary to consider several limitations associated with this approach, primarily stemming from the inability of *in vitro* studies to accurately replicate the complex *in vivo* environment. Such limitations hinder a comprehensive understanding of the true behavior and therapeutic potential of stromal and vascular cells and ADSCs. The authors do not anticipate any specific regulatory or cost advantages of Liporevive compared to existing devices, as this was not within the scope of the current study.

## Conclusions

Liporevive is a simple technique that allows obtaining nanofat without significant DNA reduction compared to lipoaspirate and microfat, and contains stromal, vascular and adipose-derived stem cells. This study was conducted *in vitro*, providing valuable preliminary insights; however, further *in vivo* and clinical validation is essential to confirm the findings and ensure their safety, efficacy, and reliability before Liporevive can be adopted for routine clinical use.

## Funding

None.

## Ethical approval statement

The protocol was conducted in accordance with the World Medical Association Declaration of Helsinki “Ethical Principles for Conducting Medical Research Involving Human Subjects” (rev. in 2013) and the Rules of Good Clinical Practice in the Russian Federation (June 19, 2003, Registration No 266). The protocol of the study was approved by Ethics Board of VIP Clinic (Approval letter, October 12, 2023). Informed consents were received from all participants prior to inclusion in the study.

## Declaration of competing interest

None.

## Appendix Supplementary materials

Image, image 1

Online Supplementary Material 1. Unmodified Figure 3. Image, application 2
