# Supplementary figures and images for: Improvement in nanofat preparation technology: Simple and easy-to-use adipose tissue harvesting with Liporevive
Source: JPRAS Open. 2025 Sep 21;46:187–99. doi: 10.1016/j.jpra.2025.09.019 (PMC12596517; doi:10.1016/j.jpra.2025.09.019)

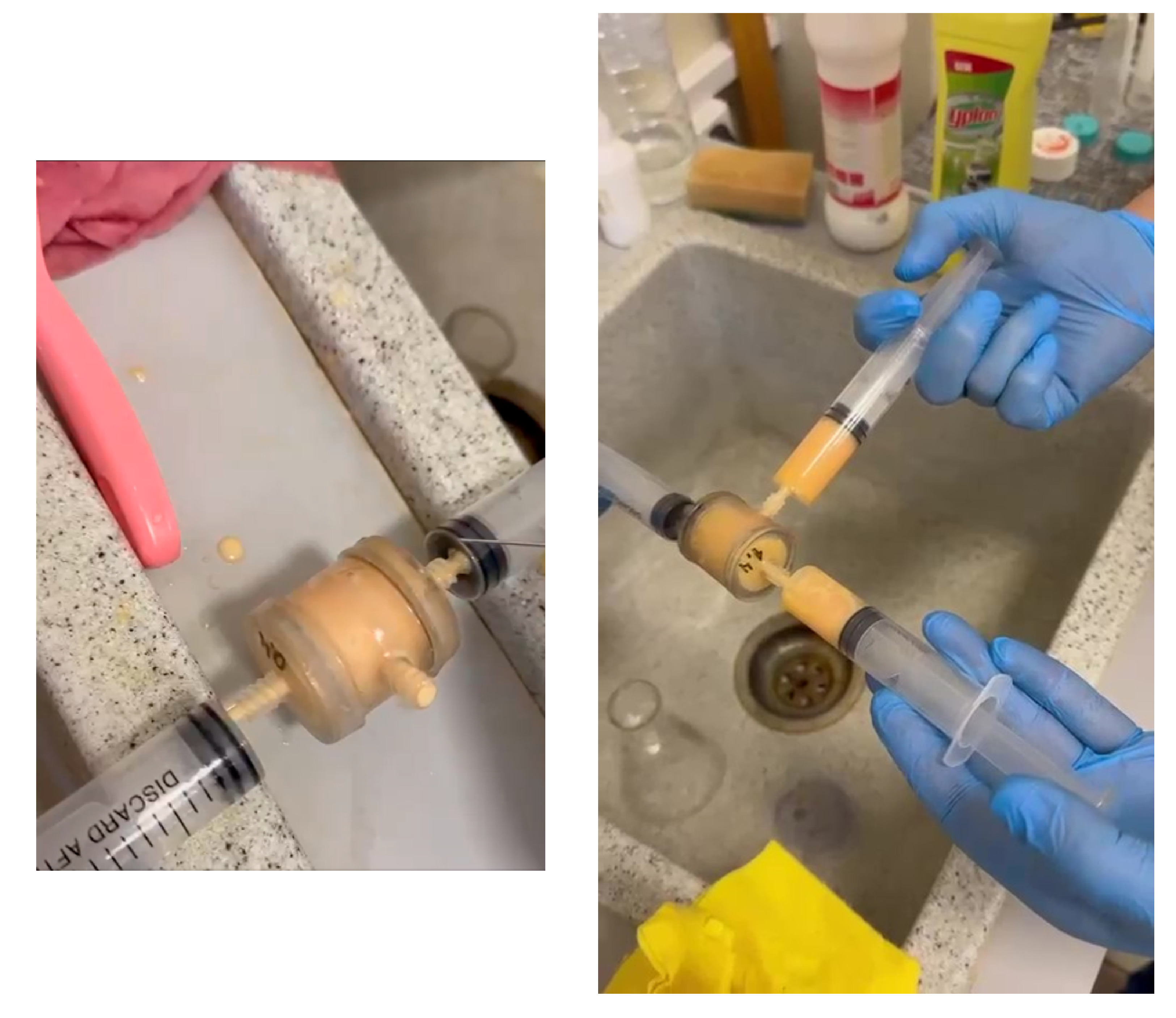

Supplement: Supplementary file 1 [file mmc1.jpg]
