# Supplementary material for: Improvement in nanofat preparation technology: Simple and easy-to-use adipose tissue harvesting with Liporevive
Source: JPRAS Open. 2025 Sep 21;46:187–99. doi: 10.1016/j.jpra.2025.09.019 (PMC12596517; doi:10.1016/j.jpra.2025.09.019)
Supplement: Supplementary file 2 — Online Supplementary Material 1. Unmodified Figure 3. [file mmc2.docx]

**Supplementary materials 2.**

**2. Materials and methods**

**Detailed description of the Samples**

•S1 – non-processing lipoaspirate.

•S2 – lipoaspirate, sequentially passed 30 times through Luer-to-Luer Syringe adapter (D-1.4 mm). S2 corresponded to microfat.

•S3 – lipoaspirate, sequentially passed 30 times through mesh with pore size 1.4 mm using 20-cc syringes and 30 times through mesh with pore size 0.6 mm using 20-cc syringes by Liporevive. S3 was obtained from fraction 0.6 mm and corresponded to nanofat.

•S4 – lipoaspirate, sequentially passed 30 times through mesh with pore size 1.4 mm using 20-cc syringes and 30 times through mesh with pore size 0.4 mm using 20-cc syringes by Liporevive. S4 was obtained from fraction 0.4 mm and corresponded to nanofat.

•S5 – lipoaspirate, sequentially passed 30 times through mesh with pore size 0.8 mm using 20-cc syringes and 30 times through mesh with pore size 0.6 mm using 20-cc syringes by Liporevive. S5 was obtained from fraction 0.6 mm and corresponded to nanofat.

•S6 – lipoaspirate, sequentially passed 50 times through mesh with pore size 1.4 mm using 20-cc syringes and 50 times through mesh with pore size 0.6 mm using 20-cc syringes by Liporevive. S6 was obtained from fraction 0.6 mm and corresponded to nanofat.

•S7 – lipoaspirate, sequentially passed 50 times through mesh with pore size 1.4 mm using 20-cc syringes and 50 times through mesh with pore size 0.4 mm using 20-cc syringes by Liporevive. S7 was obtained from fraction 0.4 mm and corresponded to nanofat.

The emulsified adipose tissue appears whitish; all Samples were then processed for comparative analysis; nanofat Samples S3-S7 appeared homogeneous fat emulsions.

The obtained Samples were then divided into 2 parts. From the first part, unfixed Samples’ smears (S2-S7) were prepared by applying Samples onto Thermo Scientific™ SuperFrost Plus© slides. After drying smears were fixed in 10% formalin and stained as described below. The second part of the Samples (S1-S7) were fixed in 10% formalin in a ratio of 1:2 (Samples:Formalin), embedded in paraffin, prepared 2-micrometers thickness sections and stained as described below.

**2.2. Immunohistochemistry and Microscopy**

Formalin-fixed, paraffin-embedded 2 µm sections of Samples S1-S7 were initially deparaffinized and subsequently rehydrated through a gradual reduction in methanol concentration. Slides with unfixed Samples’ smears S2-S7 were fixed by 10% formalin solution and rinsed in PBS for 5 minutes. To facilitate antigen retrieval, Trilogy buffer (Sigma-Aldrich, Germany) was employed according to the manufacturer's protocol.

To block endogenous peroxidase activity, a 3% hydrogen peroxide solution was applied for 20 minutes. To prevent nonspecific binding, a 1% BSA/PBS block solution was used for 30 minutes at room temperature. Primary antibodies targeting Von Willebrand factor (vWF) (Abcam, cat #ab6994), ɑ-Smooth muscle actin (ɑ-SMA) (Abcam, cat #ab5694) or CD73 for Adipose-derived stem cells (ADSCs, Abcam, ab175396) applied to the slides at a 1:100 dilution in block solution and incubated for 1 hour at room temperature. Peroxidase-conjugated Real EnVision secondary antibodies (Dako, USA) were applied for 30 minutes.

Following staining, three PBS washes were conducted for 5 minutes each. HiDef Detection™ HRP Polymer Detector (Cell marque, USA) was then applied for 10 minutes. The DAB chromogen substrate (DAKO, USA) was applied for 2 minutes, with the reaction being blocked in distilled water. The slides were dehydrated in graded methanol baths and xylene and were ultimately mounted using Cytoseal™ 60 (Thermo Scientific). The staining was supplemented with hematoxylin and eosin (H&E) according to standard protocol.

Scanning of the slides was performed on Aperio ImageScope (v12.4.3.5008, Leica Microsystems GmbH).

**2.3. Lipids staining and Microscopy**

Unfixed Samples’ smears (S2-S7) were obtained for lipids by Sudan III staining. Briefly, Samples were fixed by 10% formalin solution, rinsed by PBS, dehydrated by 70% ethanol for 10 minutes and thereafter incubated for 30 minutes in an alcohol solution of Sudan III (Biovitrum, Russia). Slides were successively washed by 70% ethanol for 10 minutes and PBS, supplemented with hematoxylin and eosin (H&E) for visualization and were ultimately mounted using Aqua-Poly/Mount (PolySciences). The images were obtained by Aperio ImageScope (v12.4.3.5008, Leica Microsystems GmbH).

To quantify the area stained for Sudan III, the Positive Pixel Count v9 algorithm was employed, which quantifies pixels based on predefined color, intensity, and saturation criteria. Initial algorithm parameters were configured to differentiate between positive (orange) and negative (other colors) pixels. The algorithm's output included the count of positive pixels (Np) and negative pixels (Nn). Subsequently, a staining score was calculated by the algorithm as Np / (Np + Nn).

**2.4. Evaluation of DNA quantity in Samples**

Samples were transferred into Eppendorf tubes, prefilled with beads (Retsch, Germany), combined with equal amounts of lysis buffer (4% SDS, 500 mM NaCl, 50 mM Tris-HCL (pH 8.0) and 50 mM EDTA). Samples were incubated for 10 min at 900C, homogenized for 4 minutes at 3000 rpm, heated for 20 min at 900C and centrifuged at 12000 g for 15 min. 600 µl of each Sample were combined with 600 µl of chloroform/isoamyl alcohol (24:1) mixture vortexed for 30 sec and centrifuged for 20 min at 16000 g. 500 µl of contained DNA supernatant was mixed with 50 µl 5M NaCl and 1250 µl 96% ethanol and incubated at -200C overnight. Samples were centrifuged at 16000 g for 20 min at -40C; pellets were washed with 500 µl of 80% ethanol and dehydrated at 370C for 15 minutes. «Qubit
